# Supplementary material for: Highly Stable, Mechanically Enhanced, and Easy-to-Collect Sodium Alginate/NZVI-rGO Gel Beads for Efficient Removal of Cr(VI)
Source: Polymers (Basel). 2023 Sep 14;15(18):3764. doi: 10.3390/polym15183764 (PMC10534353; doi:10.3390/polym15183764)
Supplement: Supplementary file 1 [file polymers-15-03764-s001.zip › polymers-2601282-supplementary.pdf]

Supplementary Information

Highly Stable, Mechanically Enhanced, and Easy-to-Collect Sodium Alginate/NZVI-rGO Gel Beads for Efficient Removal of Cr(VI)

Qi Jing *, Yuheng Ma, Jingwen He and Zhongyu Ren

Faculty of Architecture, Civil and Transportation Engineering, Beijing University of Technology, Beijing 100124, China
* Correspondence: jingqi@bjut.edu.cn; Tel.: +86-185-0195-00178

Text S1. Swelling ratio and water content

The swelling ratio (SR%) was determined by immersing the dried SA/NZVI-rGO beads in aqueous solutions of different pH at room temperature until reaching equilibrium and the SR was calculated using Eq. (S1).

$$SR = \frac{W_s - W_d}{W_d} \qquad \text{(S1)}$$

where W_s is the bead weights after swelling and W_d is the bead weights in the dry state.

The water content (WC%) of SA/NZVI-rGO hydrogel beads was calculated by weighing them after freeze-drying using Eq. (S2).

$$WC = \frac{W_0 - W_d}{W_0} \times 100\% \qquad \text{(S2)}$$

where W_0 is the wet bead weights and W_d is the dry bead weights.

Text S2. The calculation of removal capacity and removal efficiency

The removal capacity (mg/g) was calculated using Eq. (S3).

$$q_t = \left(C_0 - C_t\right)\frac{V}{m} \qquad \text{(S3)}$$

where C_0 and C_t are the initial concentration and the concentration after a period t (mg/L), respectively; V is the initial solution volume (L); and m is the adsorbent dosage (g).

The removal efficiency of Cr(VI) is calculated by Eq. (S4).

$$Removal(\%) = \frac{\left(C_0 - C_t\right)}{C_0} \times 100 \quad \text{(S4)}$$

where C_0 and C_t are the initial concentration and the concentration after a period t (mg/L), respectively.

Text S3. Reusability of SA/NZVI-rGO gel beads

Adsorbents that can be easily recovered decrease the risk of secondary pollution to the environment, and the recovery and reuse of adsorbents are important factors in

reducing costs and increasing efficiency. Therefore, the SA/NZVI-rGO beads were desorbed by shaking in a water-bath thermostatic oscillator for 1 h with 0.01 M HCl solution and washed several times with deionized water to remove the surface H^+. The treated gel beads were re-applied and the operation was repeated several times. Fig S5 shows that the removal efficiency and removal capacity could reach 87.73 % and 3.59 mg/g for the second use, but the removal efficiency decreased to 53.27% and 28.08% for the third and fourth use, respectively, which could be attributed to the repeated oxidation reaction of the gel beads with the highly oxidizing Cr (VI) breaking the polymer chains and thus leading to the reduction of the active sites(Sarojini et al., 2021). SA/NZVI-rGO beads could be effectively utilized three times.

Text S4. Theoretical column models of Thomas model, Adam-Bohart model and Yoon-Nelson model

Thomas model supposes that a flat push flow effect in the fixed bed removal process(Thomas, 1948), and the model equation is explained by Eq. (S5) below.

$$\frac{C_t}{C_0}=\frac{1}{1+\exp\left(K_T q_0 \frac{m}{Q}-K_T C_0 t\right)} \quad \text{(S5)}$$

where C_0 is the influent concentration (mg/L), C_t is the effluent concentration (mg/L), K_T is the Thomas model constant (L/mg/min), q_0 is the maximum removal capacity (mg/g), m is the gel beads mass in the column (g) and Q is the flow rate (L/min).

Adam-Bohart model was derived from surface theory which provides the dynamic relationship between C_t/C_0 in column mode, the model can predict the relationship between bed depth and breakthrough occurrence time (Bohart and Adams, 1920), and the model can be expressed using Eq. (S6).

$$\frac{C_t}{C_0}=\frac{1}{1+\exp(K_{AB} N_0 \frac{h}{v}-K_{AB} C_0 t)} \quad \text{(S6)}$$

where C_0 is the influent concentration (mg/L), C_t is the effluent concentration (mg/L), K_{AB} is the kinetic constant (L/mg/min), N_0 is the saturation concentration (mg/L), h is the bed depth (cm), and v is the linear velocity (cm/min) calculated by dividing the flow rate of the column by the cross-sectional area.

Yoon-Nelson model is a semi-empirical model, which is fitted without considering the flow rate and adsorbent dosage, requiring fewer known parameters in a simple form (Yoon and Nelson, 1984). The model is expressed by the following Eq. (S7).

$$\frac{C_t}{C_0}=\frac{1}{1+\exp(K_Y t-\tau K_Y)} \quad \text{(S7)}$$

where K_Y is rate constant (min^{-1}), τ is the time required for 50% removal breakthrough (min).

Table S1. Comparison of the removal efficiency of Cr(VI) by NZVI-based materials under alkaline conditions.

Adsorbent	pH range	Minimum removal efficiency (%)	Reference
P-BC/nZVI	2-11	62.82	(Yi et al., 2023)
C-nZVI@BC	2-8	35.22	(Wang et al., 2023)
S-nZVI@H	4-8	34.2	(Tian et al., 2023)
Fe@LDH/rGO	3-11	29.8	(Lv et al., 2019)

nZVI/graphene composites	2-12	24	(Phyu Mon et al., 2023)
nZVI@LH	2.1-10.1	18.5	(Liu et al., 2022)
SA/NZVI-rGO gel beads	3-11	82.1	Present study

Table S2. Composition of the actual Cr(VI) wastewater before and after treatment.

Composition	Before treatment (mg/L)	After treatment (mg/L)
Cr(VI)	13.75	4.09
Cl^-	1462.9512	1091.7250
NO_3^-	48.8570	40.3813
SO_4^{2-}	13.7516	28.0062
PO_4^{3-}	51.2167	45.1446
Ca^{2+}	110.084	191.8199
Cu^{2+}	0.0013	0.0009
K^+	314.3645	300.0982
Mg^{2+}	29.8242	24.4651
Na^+	588.8296	627.5543
Zn^{2+}	0.0011	0.0026

Table S3. Comparison of maximum adsorption capacity of Cr(VI) by NZVI-based materials.

Adsorbent	Removal capacity (mg/g)	Reference
Cit-nZVI@BC	99.73	(Zhou et al., 2022)
AVT-nZVI	59.17	(Zhao et al., 2019)
P-BC/nZVI composite	54.8	(Yi et al., 2023)
NZVI/carbon/alginate composite gel	35.25	(Wen et al., 2020)
$CaCO_3$-nZVI	31.76	(Cheng et al., 2021)
Fe-SA-C900 hydrogel	27.9	(Zhao et al., 2021)
S-nZVI@H	9.24	(Tian et al., 2023)
SA/NZVI-rGO gel beads	55.42	Present study

Table S4. Effect of bed height on column adsorption model constants.

	Thomas			Adam-Bohart			Yoon-Nelson		
Bed height (cm)	K_T (L/mg/min) (10^-3)	q_0 (mg/g)	R^2	K_{AB} (L/mg/min) (10^-3)	N_0 (mg/L)	R^2	K_{YN} (min^{-1}) (10^-3)	τ (min)	R^2
4.5	0.178	2.558	0.925	0.178	138.537	0.925	-1.81	230	0.887
6.5	0.141	5.798	0.974	0.415	307.944	0.974	-1.42	1134	0.974
8.5	0.977	7.484	0.942	0.974	396.644	0.942	-1.03	1582	0.919

Table S5. Effect of initial concentration on column adsorption model constants.

	Thomas			Adam-Bohart			Yoon-Nelson		
influent concen-tration (mg/L)	KT (L/mg/min) (10^-3)	q_0 (mg/g)	R^2	K_{AB} (L/mg/min) (10^-3)	N_0 (mg/L)	R^2	K_{YN} (min^{-1}) (10^-3)	τ (min)	R^2
10	0.977	7.484	0.942	0.977	396.653	0.942	-1.03	1582	0.919
30	0.479	10.655	0.913	0.479	567.720	0.913	-1.46	605	0.827
40	0.431	9.715	0.933	0.431	514.909	0.933	-1.74	323	0.823

Table S6. Effect of flow rate on column adsorption model constants.

	Thomas			Adam-Bohart			Yoon-Nelson		
Flow rate (ml/min)	K_T (L/mg/min) (10^-3)	q_0 (mg/g)	R^2	K_{AB} (L/mg/min) (10^-3)	N_0 (mg/L)	R^2	K_{YN} (min^{-1}) (10^-3)	τ (min)	R^2

0.7	0.186	2.400	0.925	0.178	138.537	0.925	-1.94	230	0.888
2	0.41	0.370	0.905	0.410	20.257	0.905	-4.10	16.13	0.905
4	0.428	0.571	0.827	0.478	27.811	0.836	-4.77	11.12	0.836

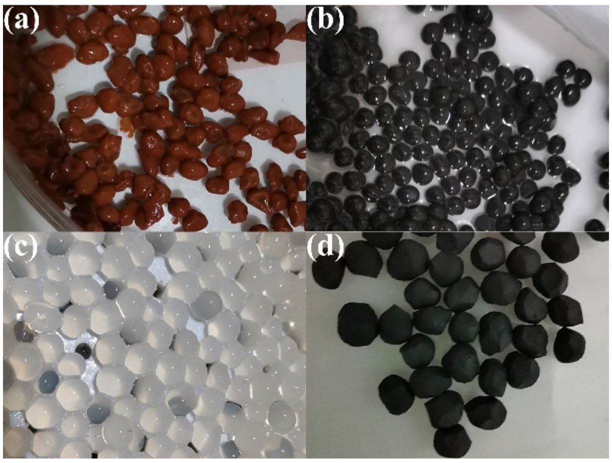

Figure S1. Photographs of (a) SA/NZVI beads and (b) SA/NZVI-rGO beads stored under seal for 226 days; Photographs of (c) SA beads and (d) freeze-dried SA/NZVI-rGO beads.

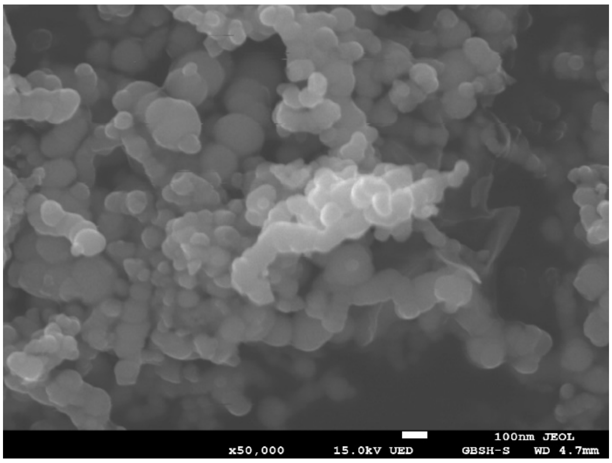

Figure S2. SEM of NZVI.

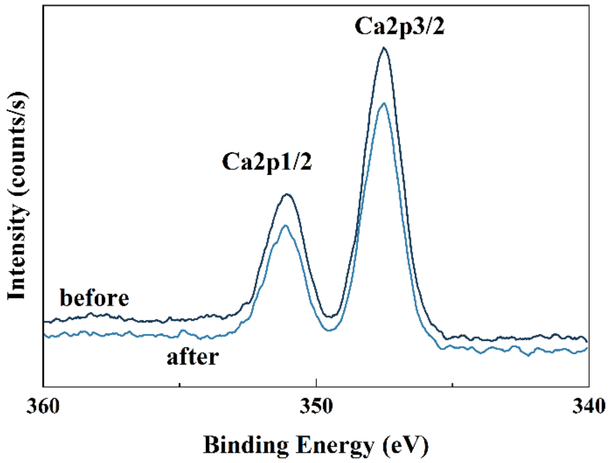

Figure S3. Ca 2p XPS spectra of SA/NZVI-rGO beads before and after removal Cr(VI).

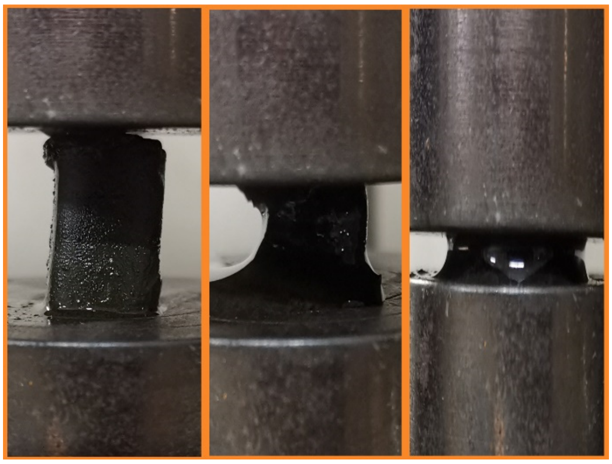

Figure S4. Photographs of hydrogels before, during, and after compression.

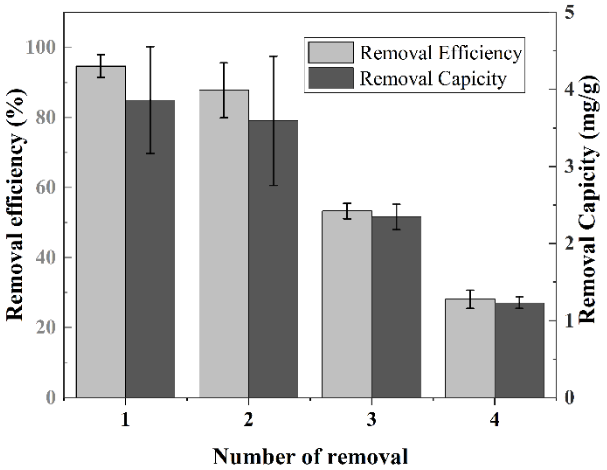

Figure S5. The reuse of SA/NZVI-rGO gel beads (initial concentration 20mg/L, pH3, dosage 0.5g, 298K).

References

1. Bohart, G.S., Adams, E.Q., 1920. SOME ASPECTS OF THE BEHAVIOR OF CHARCOAL WITH RESPECT TO CHLORINE.1. J. Am. Chem. Soc. 42, 523–544. https://doi.org/10.1021/ja01448a018
2. Cheng, Y., Dong, H., Hao, T., 2021. CaCO3 coated nanoscale zero-valent iron (nZVI) for the removal of chromium(VI) in aqueous solution. Sep. Purif. Technol. 257, 117967. https://doi.org/10.1016/j.seppur.2020.117967
3. Liu, X., Zhang, S., Zhang, X., Guo, H., Cao, X., Lou, Z., Zhang, W., Wang, C., 2022. A novel lignin hydrogel supported nZVI for efficient removal of Cr(VI). Chemosphere 301, 134781. https://doi.org/10.1016/j.chemosphere.2022.134781
4. Lv, X., Qin, X., Wang, K., Peng, Y., Wang, P., Jiang, G., 2019. Nanoscale zero valent iron supported on MgAl-LDH-decorated reduced graphene oxide: Enhanced performance in Cr(VI) removal, mechanism and regeneration. J. Hazard. Mater. 373, 176–186. https://doi.org/10.1016/j.jhazmat.2019.03.091
5. Phyu Mon, P., Phyu Cho, P., Chanadana, L., Ashok Kumar, K.V., Dobhal, S., Shashidhar, T., Madras, G., Subrahmanyam, Ch., 2023. Bio-waste assisted phase transformation of Fe3O4/carbon to nZVI/graphene composites and its application in reductive elimination of Cr(VI) removal from aquifer. Sep. Purif. Technol. 306, 122632. https://doi.org/10.1016/j.seppur.2022.122632
6. Sarojini, G., Venkatesh Babu, S., Rajamohan, N., Senthil Kumar, P., Rajasimman, M., 2021. Surface modified polymer-magnetic-algae nanocomposite for the removal of chromium- equilibrium and mechanism studies. Environ. Res. 201, 111626. https://doi.org/10.1016/j.envres.2021.111626
7. Tian, Z., Jing, Q., Qiao, S., You, W., 2023. Encapsulation/capture of S-nZVI particles by PANa-PAM hydrogel limits their leakage and improves Cr(VI) removal. Process Saf. Environ. Prot. 172, 124–135. https://doi.org/10.1016/j.psep.2023.01.068
8. Wang, T., Sun, Y., Bai, L., Han, C., Sun, X., 2023. Ultrafast removal of Cr(VI) by chitosan coated biochar-supported nano zero-valent iron aerogel from aqueous solution: Application performance and reaction mechanism. Sep. Purif. Technol. 306, 122631. https://doi.org/10.1016/j.seppur.2022.122631
9. Wen, R., Tu, B., Guo, X., Hao, X., Wu, X., Tao, H., 2020. An ion release controlled Cr(VI) treatment agent: Nano zero-valent iron/carbon/alginate composite gel. Int. J. Biol. Macromol. 146, 692–704. https://doi.org/10.1016/j.ijbiomac.2019.12.168

10. Yi, Y., Wang, X., Zhang, Y., Yang, K., Ma, J., Ning, P., 2023. Formation and mechanism of nanoscale zerovalent iron supported by phosphoric acid modified biochar for highly efficient removal of Cr(VI). Adv. Powder Technol. 34, 103826. https://doi.org/10.1016/j.apt.2022.103826
11. Yoon, Y.H., Nelson, J.H., 1984. Application of gas adsorption kinetics. I. A theoretical model for respirator cartridge service life. Am. Ind. Hyg. Assoc. J. 45, 509–516. https://doi.org/10.1080/15298668491400197
12. Zhao, C., Hu, L., Zhang, C., Wang, S., Wang, X., Huo, Z., 2021. Preparation of biochar-interpenetrated iron-alginate hydrogel as a pH-independent sorbent for removal of Cr(VI) and Pb(II). Environ. Pollut. 287, 117303. https://doi.org/10.1016/j.envpol.2021.117303
13. Zhao, R., Zhou, Z., Zhao, X., Jing, G., 2019. Enhanced Cr(VI) removal from simulated electroplating rinse wastewater by amino-functionalized vermiculite-supported nanoscale zero-valent iron. Chemosphere 218, 458–467. https://doi.org/10.1016/j.chemosphere.2018.11.118
14. Zhou, H., Ma, M., Zhao, Y., Baig, S.A., Hu, S., Ye, M., Wang, J., 2022. Integrated green complexing agent and biochar modified nano zero-valent iron for hexavalent chromium removal: A characterisation and performance study. Sci. Total Environ. 834, 155080. https://doi.org/10.1016/j.scitotenv.2022.155080
